# Gene Selection in Arthritis Classification With Large-Scale Microarray Expression Profiles

**DOI:** 10.1002/cfg.264

**Published:** 2003-04

**Authors:** Naijun Sha, Marina Vannucci, Philip J. Brown, Michael K. Trower, Gillian Amphlett, Francesco Falciani

**Affiliations:** 1 Mathematical Sciences Department University of Texas at El Paso El Paso TX 79968-0514 USA; 2 Department of Statistics Texas A&M University 3143 TAMU College Station TX 77843-3143 USA; 3 Institute of Mathematics and Statistics University of Kent at Canterbury Kent Canterbury CT2 7NF UK; 4 Genomics and Proteomic Sciences Division GlaxoSmithKline Medicines Research Centre Stevenage UK; 5 Statistical Sciences GlaxoSmithKline Medicines Research Centre Stevenage UK; 6 School of Biosciences University of Birmingham Birmingham B15 2TT UK

## Abstract

The use of large-scale microarray expression profiling to identify predictors of disease class has become of major interest. Beyond their impact in the clinical setting (i.e. improving diagnosis and treatment), these markers are also likely to provide clues on the molecular mechanisms underlining the diseases. In this paper we describe a new method for the identification of multiple gene predictors of disease class. The method is applied to the classification of two forms of arthritis that have a similar clinical endpoint but different underlying molecular mechanisms: rheumatoid arthritis (RA) and osteoarthritis (OA). We aim at both the classification of samples and the location of genes characterizing the different classes. We achieve both goals simultaneously by combining a binary probit model for classification with Bayesian variable selection methods to identify important genes.We find very small sets of genes that lead to good classification results. Some of the selected genes are clearly correlated with known aspects of the biology of arthritis and, in some cases, reflect already known differences between RA and OA.


**Comparative and Functional Genomics**
*Comp Funct Genom* 2003; **4**: 171–181.
Published online 1 April 2003 in Wiley InterScience (www.interscience.wiley.com). **DOI:** 10.1002/cfg.264

**Primary Research**

### Gene selection in arthritis classification with large-scale microarray expression profiles

Naijun Sha[1], Marina Vannucci[2]*, Philip J. Brown[3], Michael K. Trower[4], Gillian Amphlett[5] and Francesco Falciani[6]

[1] *Mathematical Sciences Department, University of Texas at El Paso, El Paso, TX 79968-0514, USA*
[2] *Department of Statistics, Texas A&M University, 3143 TAMU, College Station, TX 77843-3143, USA*
[3] *Institute of Mathematics and Statistics, University of Kent at Canterbury, Canterbury, Kent CT2 7NF, UK*
[4] *Genomics and Proteomic Sciences Division, GlaxoSmithKline, Medicines Research Centre, Stevenage, UK*
[5] *Statistical Sciences, GlaxoSmithKline, Medicines Research Centre, Stevenage, UK*
[6] *School of Biosciences, University of Birmingham, Birmingham B15 2TT, UK*

**Correspondence to:*
*Marina Vannucci, Department of Statistics, Texas A&M University, 3143 TAMU, College Station, TX 77843-3143, USA.*
*E-mail: mvannucci@stat.tamu.edu*

Received: 2 August 2002
Revised: 18 January 2003
Accepted: 30 January 2003

### Abstract

**The use of large-scale microarray expression profiling to identify predictors of disease class has become of major interest. Beyond their impact in the clinical setting (i.e. improving diagnosis and treatment), these markers are also likely to provide clues on the molecular mechanisms underlining the diseases. In this paper we describe a new method for the identification of multiple gene predictors of disease class. The method is applied to the classification of two forms of arthritis that have a similar clinical endpoint but different underlying molecular mechanisms: rheumatoid arthritis (RA) and osteoarthritis (OA). We aim at both the classification of samples and the location of genes characterizing the different classes. We achieve both goals simultaneously by combining a binary probit model for classification with Bayesian variable selection methods to identify important genes. We find very small sets of genes that lead to good classification results. Some of the selected genes are clearly correlated with known aspects of the biology of arthritis and, in some cases, reflect already known differences between RA and OA. Copyright © 2003 John Wiley & Sons, Ltd.**

**Keywords:** **Bayesian variable selection; classification; gene expression profiling**

### Introduction

Diagnostics and clinical classification is traditionally based on the integrated use of clinical, histological and biochemical information. Molecular markers that are informative of a disease are the result of years of clinical research and often presuppose understanding of the molecular pathology of the disease. Recently, microarray technology has provided clinical researchers with a powerful tool that allows the screening of thousands of genes in single experiments. This unprecedented amount of information has made possible a detailed analysis of the transcriptional state of a diseased tissue. Such screenings may not require previous hypotheses, since appropriate statistical methods can be applied to discover associations between the expression of informative genes and disease status. These associations can generate hypotheses on the molecular mechanisms behind the different diseases. This approach has already been very successful. Clustering (Eisen *et al*., 1998; Alizadeh *et al*., 2000), classical regression techniques (Nguyen *et al*., 2002), neural networks (Khan *et al*., 2001) and other methods (Golub *et al*., 1999; Dopazo *et al*., 2001) have been successfully applied to microarray data for classification purposes. Although the field is clearly very promising, the classification of clinical samples by microarrays is far from being a standard procedure. The problem is particularly challenging for statisticians, in that microarray data usually consist of many correlated variables (gene

Copyright © 2003 John Wiley & Sons, Ltd.

expression measurements) and relatively few samples. The large number of genes measured in every experiment makes the selection of the really informative variables a difficult task. Models that are highly predictive but use too many variables may be impractical in clinical practice and may be difficult to interpret. Moreover, some of the available methods (e.g. based on simple correlation or distance measures) tend to include highly correlated genes in the model. These models can lead to good predictors but they may not fully represent the biological complexity of the disease, hence the need to optimize both accuracy and complexity of the models. Here we describe a method that specifically addresses this issue. We model the expression data through a binary probit model for classification and use Bayesian variable selection methods (Brown, Vannucci and Fearn, 1998) to select important genes. Our model assigns a posterior probability to every possible subset of the genes and then proceeds by searching for those subsets that have high posterior probability. This results in the location of multiple genes that characterize the different classes. Marginal posterior probabilities of single genes are also computed. The readers are referred to Shoemaker *et al.* (1999) for a gentle introduction to Bayesian methods in genetics.

We apply our methodology to a problem of immunological interest: the classification of two clinically distinct forms of arthritis, rheumatoid arthritis (RA) and osteoarthritis (OA). Although the two forms of the disease reach the same clinical endpoint of joint destruction, the aetiology and pathogenesis of the diseases are quite distinct (Konig *et al.*, 2000). RA is a systemic disease characterized by an aggressive infiltration of the synovium by activated leucocytes and the production of an invasive cell mass (pannus), which degrades cartilage and bone. OA, on the other hand, does not display these histological features, but degradative proteases are nevertheless produced in the synovium. Because of the inflammatory component that characterizes RA, the discrimination between established RA and OA is an ideal scenario to assess the significance of classification methods. Our method is able to locate small sets of genes that lead to good classification results. When compared with other methods, our approach performs well and identifies highly informative sets of genes. Some of the selected genes indeed reflect known differences in the biology of the diseases (i.e. inflammation and proliferation); some others may be linked to previously uncharacterized aspects of the disease.

### Materials and methods

### Rheumatoid arthritis and osteoarthritis: experimental study

The study was conducted on synovial tissue samples obtained from 24 RA and seven OA patients. The patients were undergoing treatment at the Norfolk and Norwich Hospital using comparable multi-agent drug therapies before being scheduled for joint arthroplasty. Synovial tissue samples were surgically removed during the joint replacement surgery and immediately frozen in dry ice. Total RNA was extracted from snap-frozen individual tissue samples using RN AzolB™ (Biogenesis, Bournemouth, UK). The mRNA was purified from total RNA using Oligotex™ mRNA Mini kit (Qiagen, Crawley, UK), according to the manufacturers instructions. Radiolabelled cDNA probes were synthesized from mRNA derived from synovial tissue samples and hybridized to nylon high-density arrays. The array was constructed in-house using 1050 cDNA clones from the publicly available IMAGE collection (Lennon *et al.*, 1996). The selected clones (representing 500–1000 bp from the 3′ end of the mRNA) were chosen to represent 755 genes of known function. The selection of these genes was not based on their involvement with the disease mechanism, but rather to represent the majority of relevant functions in the physiology of the cell. Some redundancy was introduced into this collection to control for clone-to-clone variability. Typically, 98% of the clones representing the same gene were highly correlated when expression profiles were compared ($r > 0.9$). Bacterial clones were isolated from individual colonies, grown in selection media, and the cDNA inserts PCR amplified. The quality of the amplification products was assessed by agarose gel electrophoresis and the identity of the clones verified by 3′ end sequencing. Each PCR product was then spotted four times on nylon arrays using a Qbot robot (Genetix, UK) and the filters processed. Every tissue sample was hybridized on an individual array. After hybridization, the arrays were scanned using a phosphorus imager and the resulting images were

Copyright © 2003 John Wiley & Sons, Ltd. 

processed using a proprietary image analysis package. Data were processed as follows: the mean of the logs of the four replications was derived and the value was corrected for the patient mean. The number of B lymphocytes in the tissue of the RA and OA patients was determined after staining for a B cell marker (using an anti-CD20 antibody).

### Binary probit models with latent variables

We model the expression data using a binary probit model. Let $(Z, X)$ indicate the observed data, with $X_{n\times p}$ being the predictor matrix and $Z_{n\times 1}$ being a binary response vector, coded as 0.1. In our experiment $x_{ij}$ is the expression level of gene $j$ as measured in the $i$th sample, while $z_i$ is the class label of the sample. Each outcome $z_i$ is associated with the probability $p_{i,0}$ (and consequently $p_{i,1} = 1 - p_{i,0}$) that the $i$th sample falls into the first category. A possible approach to inference in a probit model is via latent variables, transforming the model into a normal linear model on the latent responses. The key to this approach is to assume the existence of a continuous unobserved or latent variable underlying the observed categorical. When the latent variable crosses a threshold, the observed category changes.

### Bayesian framework for variable selection and posterior inference

Bayesian approaches to statistical inference impose prior distributions on the unknown parameters. We incorporate in the model a variable selection mechanism to select important genes by utilizing Bayesian mixture priors for the regression coefficients to perform the selection (Brown, Vannucci and Fearn, 1998). The key idea of the selection is to impose a mixture prior on the regression coefficients of the model that depends on a latent binary $p$-vector $\gamma$. The $j$th element of $\gamma$, $\gamma_j$, may be either 1 or 0, according to whether the $j$th variable (gene) is or is not included in the model. With $p$ genes there are $2^p$ possible models, i.e. subsets of genes. *A priori* we assume that the probability of each model is Bernoulli $\pi(\gamma) = w^{p_\gamma}(1-w)^{p-p_\gamma}$, where $p_\gamma$ indicates the number of chosen genes, i.e. the number of ones in $\gamma$.

Having set the prior distributions, a Bayesian analysis proceeds by updating the prior beliefs with the information that comes from the data. Our interest is in the posterior distribution of the vector $\gamma$, given the data. Vector values with high probability will, in fact, identify important sets of genes. Given the large number of possible vector values, i.e. $2^p$ with $p = 755$, we employ stochastic search techniques to look for subsets of genes that have high posterior probability. In brief, our method visits a sequence of models that differ successively in one or two variables (genes) by adding or deleting one variable or swapping two variables at a time. At each step the new candidate model is accepted with a probability that depends on the ratio of the relative posterior probability of the candidate model vs. the previous visited model. Details of the statistical *a posteriori* inference can be found in Sha *et al.* (2002).

### Classification of future samples

Our method allows us not only to locate sets of genes that help in characterizing the different classes but also to classify future samples. The stochastic search algorithm previously described gives us, in fact, a list of visited subsets, as well as a sample of values on the latent variable underlying the categorical variables that determine the class label of a sample. We compute conditional posterior probabilities of all distinct visited $\gamma$s. Marginal probabilities of inclusion of single genes $P(\gamma_j = 1)$, $j = 1, \ldots, p$, can be computed from the posterior probabilities of the visited models. Various prediction methods are then possible. In the experimental study described here we found good error rates by computing least squares and Bayes predictions with single models, typically the model with the highest posterior probability or the models including variables with marginal probability greater than a certain threshold. Having obtained a prediction on the latent variable, the corresponding predicted categorical value was computed.

### Analysis of the predictors and comparison with other methods

Cluster analysis was performed using an average linkage algorithm (UPGMA) on a correlation matrix obtained using the Pearson correlation coefficient. The analysis was done using the software J-express (Dysvik and Jonassen, 2001). PCA- and PLS-based discriminant analyses were performed on the training data. Both linear discriminant analysis (LDA) and quadratic discriminant analysis

Copyright © 2003 John Wiley & Sons, Ltd.

(QDA) were then applied, retaining the first components. The shrunken centroid method (Tibshirani *et al.*, 2002) was chosen as an example of an efficient method based on gene selection. The analysis with this method has been performed using the software made available by the authors. An analysis of variance (ANOVA) was performed on the genes selected in the various models to determine whether they were differentially expressed in the two classes.

### Results

### Setting up the analysis

We chose the Bernoulli prior distribution of our model (see Materials and methods) to have an expectation of 10. This means that we expect models with very few genes to perform well. We randomly split the whole data set into training and validation sets with (5,17) and (2,7) observations for the OA and RA patients, respectively. The size of the test set is roughly one-third of the original data. The chosen design allows reasonable relative sample sizes for the different classes. We fitted our model to the training data. The model assigns a posterior probability to every possible subset of the genes and then proceeds by searching for those subsets that have high posterior probability. When exploring the space of possible models, i.e. subsets, we used six parallel searches with 100 000 iterations and very different starting vectors (see Materials and methods). We discarded the first 40 000 iterations to eliminate dependence from the starting points. Starting vectors were with 1, 10, 50, 100, 150 and 300 randomly selected included genes. Each search resulted in a list of visited models with corresponding posterior probabilities. Marginal probabilities of inclusion of single genes were also computed. All methods have been implemented in Matlab and the code will be made available on the web.

### Gene selection

Figure 1 shows marginal probabilities of inclusion of single genes for the six different runs, while Figure 2 reports the marginal probabilities, recomputed by considering the union of the distinct $\gamma$s visited by the six chains. Spikes correspond to genes that have high posterior probability and that should therefore be relevant to arthritis classification. Notice how plots of Figure 1 appear overall fairly similar, despite the widely different starting values. There are also clear differences, with genes showing up in some of the plots but not in others. With so many mutually correlated variables, there are of course many good predictor sets. Our strategy can be considered satisfactory when it leads to the identification of some of these competing models.

Marginal plots like that in Figure 2 allow us to locate sets of genes that can be of interest for further investigation, simply by considering the genes with marginal posterior probability greater than a certain value. Interesting sets can be also found by exploring those models visited by the different searches that have the highest values of the posterior probability. Figure 3B (columns 1–9) provide a graphical summary of the genes that appeared to be relevant to arthritis classification. Column 1 shows the genes with marginal probability greater than 0.08. Columns 2–7 represent the single six best models found by the six different searches. Column 8 reports the overall best model, i.e. the subset with the highest posterior probability among the pooled set of distinct visited models, and in column 9 we see the genes included in the best 10 models among the pooled visited. The best 10 models accounted for 60% of the total visited probability and included 13 genes. As is to be expected, some of the genes appear to be common to more than one set in Figure 3B. In particular, selection on marginal probabilities gives a very similar set to that obtained by inspecting the best visited models. The expression profiles (heat maps) and the names of genes in the selected subsets are reported in Figure 4. The selected sets achieved very good classification results when used to predict the class label of an independent set of samples. In particular, all sets of genes that we located, displayed in Figure 3B (columns 1–9) and Figure 4, achieved a misclassification rate of 11%; this means that only 1 observation out of 9 was misclassified in the validation set. The same best error rate was found when we repeated the analysis with different splits of the data, keeping the validation set size fixed.

### Biological findings

A remarkable feature of all marginal plots in Figure 1 and in Figure 2 is the spike that corresponds to the gene with index number 290.

Copyright © 2003 John Wiley & Sons, Ltd. 

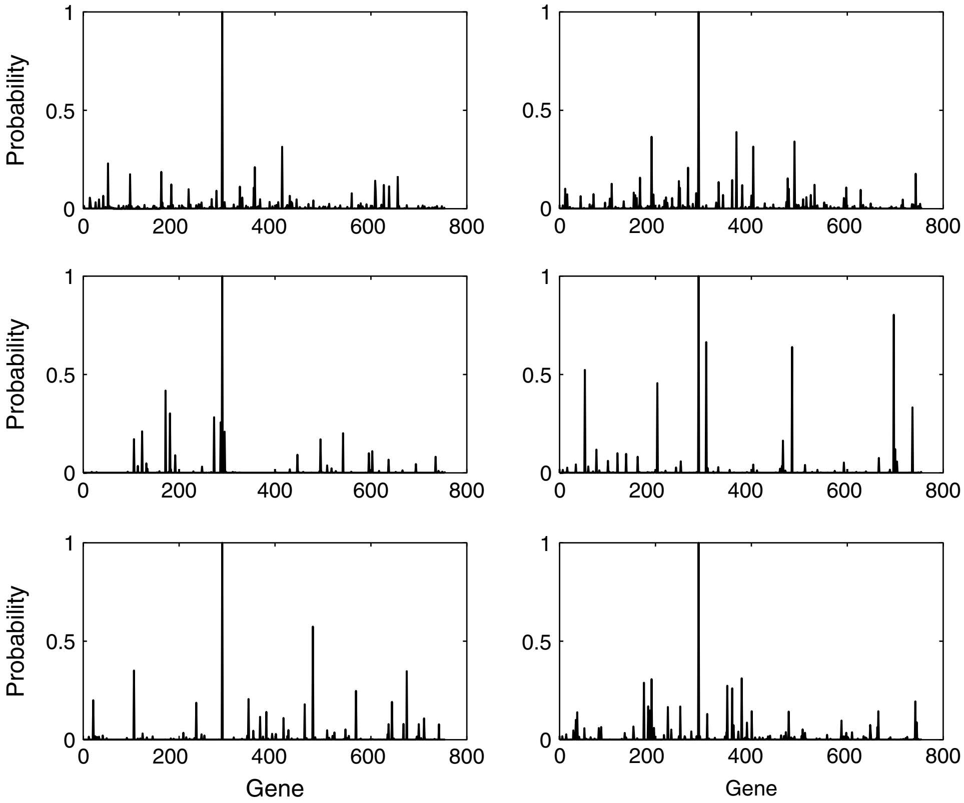

**Figure 1.** Marginal posterior probabilities of single genes for the six different runs

This is coding for a B lymphocyte-specific gene, immunoglobulin $\kappa$ heavy chain. This finding correlates well with the fact that the number of lymphocytes is on average higher in the synovial tissue of RA than in OA patients. Using an appropriate marker, we counted the number of B cells in the tissues of the two groups of patients. As expected, the number of B lymphocytes was significantly higher in RA then in OA patients (data not shown). The majority of the genes in our selected models correlate well with the disease pathology. Every predictor contains genes linked to inflammation and genes associated to other relevant pathological processes (Schett *et al.*, 2001). Examples are genes involved in proliferation (CAK, DNA replication licensing factor, proliferating cell nucleolar protein, p120), oncogenes (c-myb RHOA), adhesion (paxillin, integrin $\alpha$ 2, cytohesin, CD66), degradation (human RNA for cysteine protease, L-cathepsin) and stress response (heat shock protein 1). Selected models also contain genes linked to more specific aspects of the disease pathology, like CD47 and thrombomodulin. The first gene is associated with mechanisms of T lymphocyte activation that are particularly important in the synovial tissue of RA patients, where a large proportions of T lymphocytes are lacking CD28, a canonical co-stimulatory molecule (Vallejo *et al.*, 2000). Thrombomodulin has already been proposed as a marker for discriminating between subsets of RA patients with different disease severity (Hanyu *et al.*, 1999). Also of interest is the inclusion of the gene NRF-1. This gene encodes a transcriptional repressor involved in silencing the IFN-$\beta$ gene. It has been demonstrated that IFN-$\beta$ has immunomodulatory effects on the rheumatoid synovium cell infiltrate, altering the cytokine profile and the expression of metalloproteinase genes. It is not unreasonable to hypothesize that the NRF-silencing activity may be one of the factors contributing to the differences we observe between the two diseases. It is important to remember that further experimentation is required in order to validate these models and verify the involvement of the genes in the disease mechanisms.

Copyright © 2003 John Wiley & Sons, Ltd.

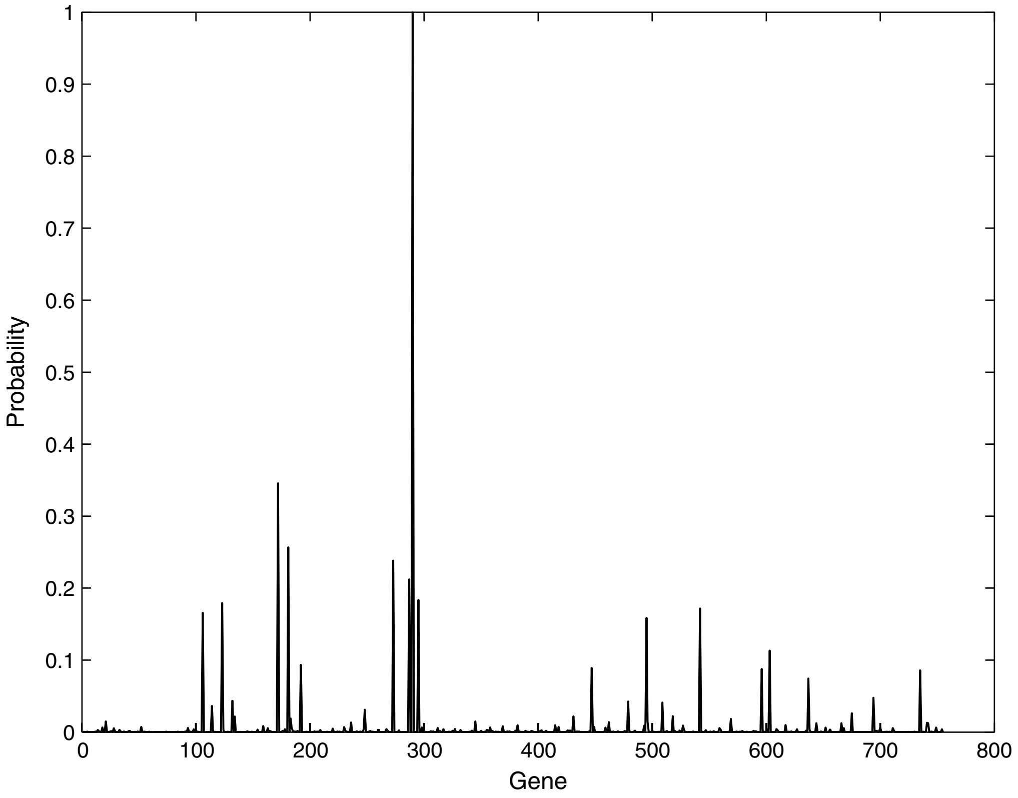

**Figure 2.** Renormalized marginal posterior probabilities of single genes computed by considering the union of the distinct subsets visited by the six chains

### Comparison with other classification methods

Our method identifies accurate predictors, but in order to assess the general validity of our approach we performed a comparison with other methods. First, we wondered whether cluster analysis would be sufficient to identify RA and OA samples as individual classes. Figure 5 represents the result of cluster analysis, with an average linkage algorithm on a similarity matrix generated by using the Pearson correlation coefficient and performed on the entire dataset. OA samples clearly tend to associate on the dendrogram but they fail to form a distinct cluster. We then tested two classical statistical techniques that use principal component analysis (PCA) to reduce the dimension of the problem. Linear discriminant analysis (LDA) and quadratic discriminant analysis (QDA) were applied after PCA, retaining the first principal components. The best results were obtained with QDA with the first five principal components and LDA with the first 13–16 components. The misclassification test error rate achieved was 22% (two out of nine) for both cases. The same error rate was found when using partial least squares (PLS) methods to reduce the dimension and retaining the first seven factors for LDA and QDA. The accuracy of these predictors is comparable with our models. Comparable classification accuracy was also obtained with a

**Figure 3.** Comparison of the selected genes in the predictive models. Panel A represents the result of the two-dimensional clustering of the arthritis datasets. The dendrogram on the left side of the gene expression map represents the clustered genes whereas the dendrogram in the upper position shows the result of clustering the samples (RA patients are represented by a blue line, OA patients are represented by a red line). Genes selected in the different models are mapped on the dendrogram in panel B. Columns 1–9 represent the models derived from our approach whereas column 10 reports the position of the genes selected in the shrunken centroid model

Copyright © 2003 John Wiley & Sons, Ltd. 

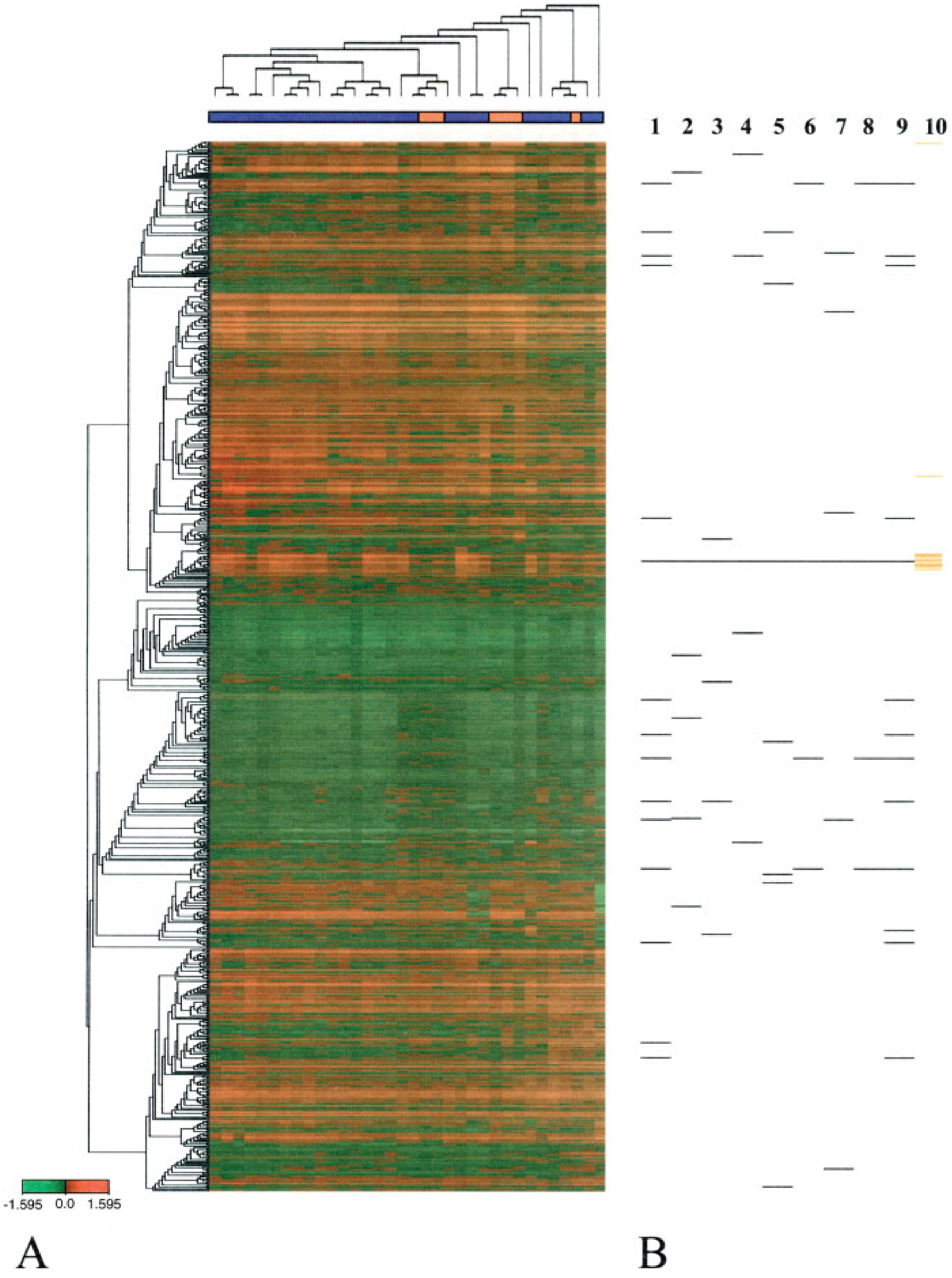

Copyright © 2003 John Wiley & Sons, Ltd.

gene selection based method recently developed by Robert Tisbshirani at the University of Stanford (Tibshirani *et al.*, 2002). The method is named 'shrunken centroid' and uses a ranking strategy for gene selection that assigns a higher score to genes whose expression is stable within samples of the same class. The selection of the genes to be included in the model is performed by a simple soft thresholding strategy. The large majority of the genes are eliminated from the predictors as the value of the threshold increases. In their approach, the authors estimate the optimal value of the parameter by cross-validation. We analysed the arthritis dataset using the programs that the authors made available. The best predictor we were able to identify was based on 12 genes (Figure 3B, column 10, and Figure 4). Its classification error was 16% (i.e. five cases out of 31 were misclassified). Notice, however, that this rate is not directly comparable with the error given by our method, achieved by splitting the data. A comparison between the genes included in our models and the 12 genes selected in the most accurate model for the shrunken centroid highlights the fact that the only feature in common is the inclusion of the gene encoding for the immunoglobulin k heavy chain. All other genes seem to differ. Interestingly, most of the genes in the shrunken centroid model are highly correlated with each other (nine out of 12), whereas the genes selected by our approach do not seem to share this property (Figure 3). Figure 4 displays the heat maps for the genes selected in the models and reports the result of an ANOVA to identify genes that are differentially expressed between the two disease classes. The results of the ANOVA prove that 11 out of 12 genes selected in the shrunken centroid method are also differentially expressed ($p < 0.01$), whereas among the genes selected in the other models it is mainly the immunoglobulin k heavy chain gene (present in all models) to be differentially regulated.

### Discussion

We have exploited a method for variable selection in classification with binary probit models. The method uses latent variables to specialize the model to a regression setting, and Bayesian mixture priors to perform the variable selection. It also employs stochastic search algorithms for posterior inference. We have shown how the method can be used to locate small sets of genes that lead to good classification errors and that are relevant for biologists to study relationships and functions. The method has been applied to the classification of two forms of arthritis that have similar clinical endpoints but different underlying molecular mechanisms: RA and OA. Selected genes that contribute to the best predictors correlate well with known aspects of the disease pathology. The most evident correlation is with the presence in RA of diseased tissues of an extensive inflammatory infiltrate, but other aspects of the pathologies also seem to be well represented. The analysis of the predictors we identified and the comparison with the model developed using the shrunken centroid approach revealed interesting properties of our method. All of the genes in the best models we identified are not correlated with each other, whereas the majority of the genes in the shrunken centroid predictor are correlated to the profile of the immunoglobulin k heavy chain gene. A possible interpretation for this observation is that the model derived from the centroid method represents a well-defined biological aspect of the disease, possibly related to the high abundance of B cells in the site of inflammation in the RA tissues. Our models definitively represent this characteristic of the disease but, also, they may model other features of the pathologies, as indicated by the inclusion of genes representing a larger spectrum of the processes involved in these pathologies (e.g. apoptosis, etc.). Another interesting property of our approach is the relatively low frequency of genes that display a differential expression between the two classes. Our strategy clearly produces models

**Figure 4.** Heat maps of the genes selected in the predictive models. Panel A displays the genes selected in the overall best model. Panels B–G display the six models obtained in the six different searches. Panel H represents the genes with marginal probability greater than 0.08 and Panel I displays the genes identified by the shrunken centroid method. A heat map represents the expression levels of each gene across the samples. On the left of the yellow line the map displays the profiles of the RA patients whereas the OA samples are on the right end side. The figure also reports the result of an ANOVA to identify genes significantly up-regulated in one of the classes. Genes marked in red are up-regulated in RA patients, genes marked in blue are up-regulated in OA patients. A threshold of $p < 0.01$ was used

Copyright © 2003 John Wiley & Sons, Ltd. 

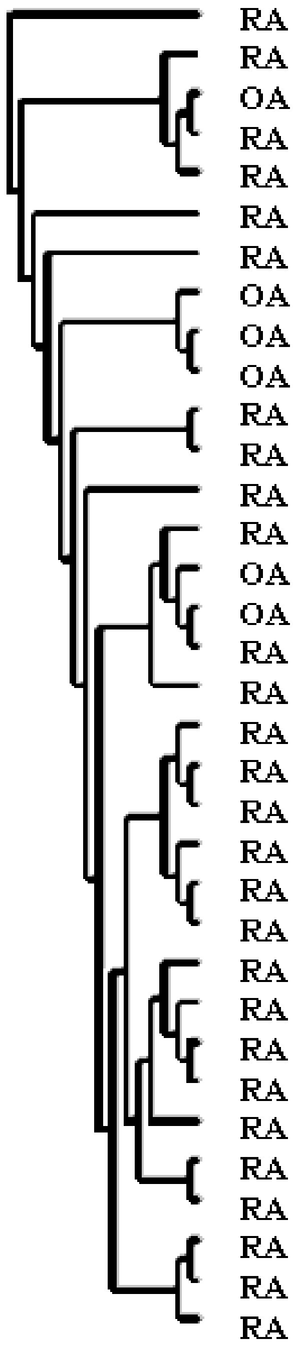

**Figure 5.** Cluster analysis of the arthritis samples. The dendrogram describes the relationships among the disease tissues. Cluster analysis was performed using an average linkage algorithm (UPGMA) on a correlation matrix obtained by using the Pearson correlation coefficient. The analysis was done using the software J-express (Dysvik and Jonassen, 2001)

with similar predictive power to conventional techniques. Interestingly, the models do not include correlated genes or a high percentage of differentially expressed genes. Extensive experimental investigation will be required to assess the biological significance of the models we described. We believe that our method is effective in linking gene expression profiles with aspects of disease pathology. The classification of subgroups of RA patients with different ability to respond to drug treatment, or at a different disease stage, will definitively be an interesting application. Such predictors would be invaluable tools in the clinic, for both prognosic and diagnostic purposes.

### Acknowledgements

We thank Ilaria Dragoni, Novartis Institute for Medical Sciences, UK, and Don M. Wallace and Tracey C. Roberts, Genomics and Proteomic Sciences Division, GlaxoSmithKline, Medicines Research Centre, Stevenage, UK. M.V. was partially supported by NSF Career Award, DMS 0093208.

### References

Alizadeh AA, Eisen MB, Davis RE, *et al.* 2000. Distinct types of diffuse large B cell lymphoma identified by gene expression profiling. *Nature* **403**: 503–511.

Brown PJ, Vannucci M, Fearn T. 1998. Multivariate Bayesian variable selection and prediction. *J R Statist Soc Ser B* **60**(3): 627–641.

Dopazo J, Zanders E, Dragoni I, Amphlett G, Falciani F. 2001. Methods and approaches in the analysis of gene expression data. *J Immunol Methods* **250**: 93–112.

Dysvik B, Jonassen I. 2001. J-Express: exploring gene expression data using Java. *Bioinformatics* **17**: 369–370.

Eisen MB, Spellman PT, Brown PO, Botstein D. 1998. Cluster analysis and display of genome-wide expression patterns. *Proc Natl Acad Sci USA* **95**: 14 863–14 868.

Golub TR, Slonim DK, Tamayo P, *et al.* 1999. Molecular classification of cancer: class discovery and class prediction by gene expression monitoring. *Science* **286**: 531–537.

Hanyu T, Arai K, Nakano M. 1999. Urinary thrombomodium in patients with rheumatoid arthritis: relationship to disease subset. *Clin Rheumatol* **18**(5): 385–389.

Khan J, Wei JS, Ringner M, *et al.* 2001. Classification and diagnostic prediction of cancers using gene expression profiling and artificial neural networks. *Nature Med* **7**(6): 673–679.

Konig A, Krenn V, Toksoy A, Gerhard N, Gillitzer R. 2000. GRO$\alpha$ and RANTES messenger RNA expression in lining layer, infiltrates and different leucocyte populations of synovial tissue from patients with rheumatoid arthritis, psoriatic arthritis and osteoarthritis. *Virchows Arch* **436**: 449–458.

Lennon G, Auffray C, Polymeropouos M, Soares MB. 1996. The I.M.A.G.E. consortium: an integrated molecular analysis of genomes and their expression. *Genomics* **33**: 151–152.

Copyright © 2003 John Wiley & Sons, Ltd. 

Nguyen DV, Rocke DM. 2002. Tumor classification by partial least squares using microarray gene expression data. *Bioinformatics* **18**: 39–50.

Schett G, Tohidast-Akrad M, Steiner G, Smolen J. 2001. The stressed synovium. *Arthritis Res* **3**(2): 80–86.

Sha N, Vannucci M, Brown PJ, Liò P. 2002. Bayesian variable selection in multinomial probit models with application to spectral data and DNA microarrays. Technical Report UKC/IMS/02/05, University of Kent at Canterbury.

Shoemaker JS, Painter IS, Weir BS. 1999. Bayesian statistics in genetics: a guide for the uninitiated. *Trends Genet* **15**(9): 354–358.

Tibshirani R, Hastie T, Narashiman B, Chu G. 2002. Diagnosis of multiple cancer types by shrunken centroids of gene expression. *Proc Natl Acad Sci USA* **99**: 6567–6572.

Vallejo AN, Mugge LO, Klimiuk PA, Weyand CM, Goronzy JJ. 2000. Central role of thrombospondin-1 in the activation and clonal expansion of inflammatory T cells. *J Immunol* **164**(6): 2947–2954.

Copyright © 2003 John Wiley & Sons, Ltd. 
